# Discoidin Domain Receptor 2 Mediates Lysophosphatidic Acid-Induced Ovarian Cancer Aggressiveness

**DOI:** 10.3390/ijms22105374

**Published:** 2021-05-20

**Authors:** Bo Young Jeong, Kyung Hwa Cho, Se-Hee Yoon, Chang Gyo Park, Hwan-Woo Park, Hoi Young Lee

**Affiliations:** 1 Department of Pharmacology, College of Medicine, Konyang University, Daejeon 35365, Korea; pharmaby@konyang.ac.kr (B.Y.J.); kh0728@konyang.ac.kr (K.H.C.); aruso@konyang.ac.kr (C.G.P.); 2 Division of Nephrology and Department of Internal Medicine, College of Medicine, Daejeon 35365, Korea; sehei@hanmail.net; 3 Department of Cell Biology, College of Medicine, Konyang University, Daejeon 35365, Korea; hwanwoopark@konyang.ac.kr

**Keywords:** lysophosphatidic acid, autotaxin, discoidin domain receptor 2, Twist1, ovarian cancer

## Abstract

Lysophosphatidic acid (LPA), a bioactive lipid produced extracellularly by autotaxin (ATX), has been known to induce various pathophysiological events, including cancer cell invasion and metastasis. Discoidin domain receptor 2 (DDR2) expression is upregulated in ovarian cancer tissues, and is closely associated with poor clinical outcomes in ovarian cancer patients. In the present study, we determined a critical role and signaling cascade for the expression of DDR2 in LPA-induced ovarian cancer cell invasion. We also found ectopic expression of ATX or stimulation of ovarian cancer cells with LPA-induced DDR2 expression. However, the silencing of DDR2 expression significantly inhibited ATX- and LPA-induced ovarian cancer cell invasion. In addition, treatment of the cells with pharmacological inhibitors of phosphoinositide 3-kinase (PI3K), Akt, and mTOR abrogated LPA-induced DDR2 expression. Moreover, we observed that HIF-1α, located downstream of the mTOR, is implicated in LPA-induced DDR2 expression and ovarian cancer cell invasion. Finally, we provide evidence that LPA-induced HIF-1α expression mediates Twist1 expression to upregulate DDR2 expression. Collectively, the present study demonstrates that ATX, and thereby LPA, induces DDR2 expression through the activation of the PI3K/Akt/mTOR/HIF-1α/Twist1 signaling axes, aggravating ovarian cancer cell invasion.

## 1. Introduction

Ovarian cancer is a deadly disease, being the fifth leading cause of cancer-associated death among women [1]. Although advanced surgical intervention and chemotherapy might improve the survival of ovarian cancer patients, the 5-year survival rate is still less than 30–50% without any specific biomarkers [2]. Ovarian cancer dissipates to various organs, such as the liver, pleura, and lungs, and the metastatic stages are critical for the survival rate of patients [3].

Lysophosphatidic acid (LPA, 1- or 2-acyl-lysophosphatidic acid) is a biolipid produced by the ectoenzyme autotaxin (ATX) [4,5,6,7]. LPA activates at least six types of G protein-coupled receptor (GPCR) to stimulate various signaling cascades for a plethora of pathophysiological events, including tumorigenesis and cancer progression [5,8,9]. Furthermore, 40 times more LPA is detected in both the plasma and the ascites of ovarian cancer patients, compared to normal human serum [10], signifying the critical role of LPA in ovarian cancer progression. LPA has been shown to induce various forms of oncogenic signaling to augment ovarian cancer progression. LPA promotes ovarian cancer invasion through a Ras/Rho/ROCK signaling pathway and through proteolytic enzyme secretion [11]. In addition, this biolipid induces HIF-1α expression to increase VEGF expression and ovarian cancer progression [12]. Furthermore, LPA was shown to induce epithelial–mesenchymal transition (EMT) by upregulating Zeb1 expression [13]. However, the detailed mechanism of the LPA-induced signaling cascade for the ovarian cancer cell invasion of the surrounding environment is still missing for the identification of valuable biomarkers to help prevent and block this deadly disease.

Located on chromosome 1q23.3, discoidin domain receptor 2 (DDR2) is a member of the receptor tyrosine kinase (RTK) family [14,15,16], and has been closely associated with the progression of various cancers, including breast and prostate cancer [17,18,19]. In addition, this collagen receptor DDR2 expression is upregulated in ovarian cancer tissues, and is associated with poor clinical outcomes in ovarian cancer patients [20,21,22]. Mechanistically, collagen1-activated DDR2 stabilizes an epithelial–mesenchymal transition transcription factor (EMT-TF) known as Snail through the Src/ERK2 signaling axis, promoting breast cancer metastasis [19]. Moreover, DDR2 was reported to induce cancer invasion via matrix metalloproteinase expression [23,24]. Furthermore, hypoxia upregulates DDR2 expression to induce Snail expression and breast cancer metastasis [25]. Meanwhile, another EMT-TF, Twist1, was recently identified as inducing DDR2 expression and ovarian cancer metastasis [21], suggesting that morphological changes by EMT-TFs might be a driving force for DDR2 expression and ovarian cancer progression.

Previously, LPA has been shown to activate EGFR for ovarian cancer cell invasion [26,27,28], suggesting transactivation between GPCR and RTK. Deciphering the important roles of another type of RTK, DDR2, in ovarian cancer progression, we hypothesize the implication of DDR2 in LPA-induced ovarian cancer progression. Therefore, we in the present study determined the role of DDR2 in LPA-induced ovarian cancer cell invasion, and found that LPA upregulates DDR2 expression through the HIF-1α/Twist1 signaling cascade to promote ovarian cancer cell invasion, expanding the underlying mechanism of LPA-induced ovarian cancer cell aggressiveness and providing potential biomarkers for ovarian cancer.

## 2. Results

### 2.1. DDR2 Is Critical for LPA-Induced Ovarian Cancer Cell Invasion

**Figure 1:**
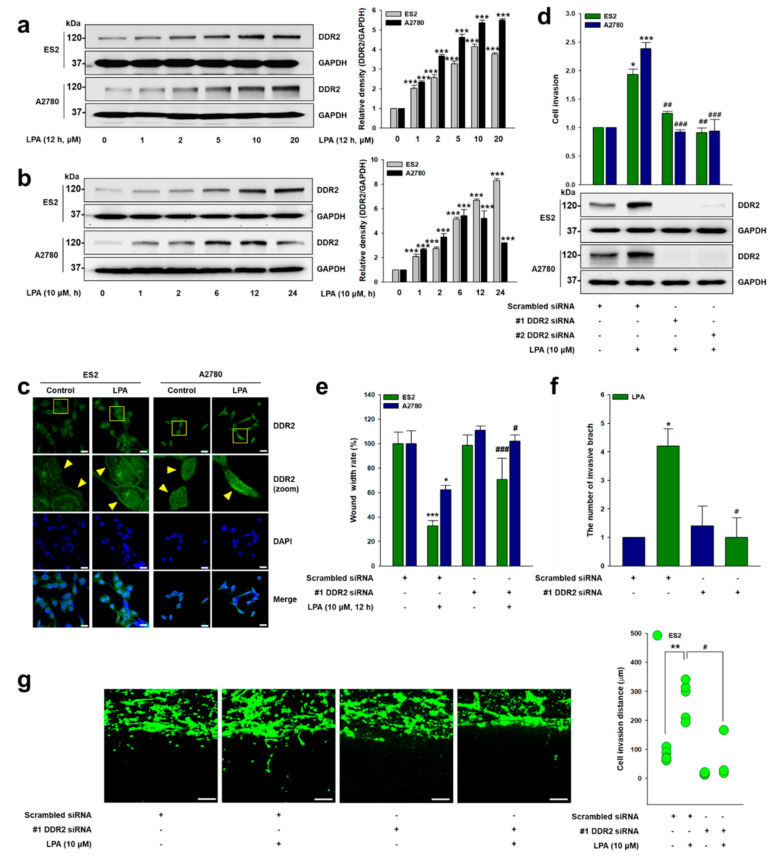
DDR2 is critical for LPA-induced ovarian cancer cell invasion. (**a**) The serum-starved cells were stimulated with LPA (12 h) at the indicated concentrations (mean ± s.d. *** *p* < 0.001 versus control). (**b**) The serum-starved cells were stimulated with LPA (10 μM) for the indicated times (mean ± s.d. *** *p* < 0.001 versus control). (**c**) The serum-starved cells were stimulated with LPA (10 μM) for 12 h, and then the expression of DDR2 was visualized via immunofluorescence (green: DDR2, and blue: Nucleus). Original magnification: ×400; scale bar: 20 μm. (**d**) The cells were transfected with indicated siRNAs, followed by stimulation with LPA (10 μM) for invasion assay (mean ± s.d. * *p* < 0.05 and *** *p* < 0.001 versus control, ## *p* < 0.01 and ### *p* < 0.001 versus LPA treatment only). (**e**) The cells were transfected with the indicated siRNAs, followed by stimulation with LPA (10 μM) for wound healing migration assay (mean ± s.d. * *p* < 0.05 and *** *p* < 0.001 versus control, # *p* < 0.05 and ### *p* < 0.001 and versus LPA treatment only). (**f**) ES2 cells were cultured on 3D Matrigel for 7 days and their branches were counted (mean ± s.d. * *p* < 0.05 versus control and # *p* < 0.05 versus LPA treatment only). (**g**) ES2 cells were transfected with the indicated siRNAs and then cultured in 3D Matrigel-coated transwell chambers. The cells were treated with LPA (10 μM), with or without DDR2 siRNA, for 5 days. The distance between the invaded cells was measured in five different positions and then calculated (mean ± s.d. ** *p* < 0.01 versus control and # *p* < 0.05 versus LPA treatment only). Original magnification: ×100; scale bar: 100 μm. Scrambled siRNAs were used as a control. Representative results were presented from at least three independent experiments with similar results.

Given that more than 40 times more LPA is observed in both the plasma and the ascites of ovarian cancer patients [10], and that DDR2 is associated with poor clinical outcomes in ovarian cancer patients [20,21], we explore the role of DDR2 in LPA-induced ovarian cancer cell invasion. Since ovarian cancer 2780 and ES2 cells express the DDR2 protein associated with ovarian cancer metastasis [21], we first stimulated these cells with LPA and observed markedly upregulated DDR2 expression in a dose-dependent manner (Figure 1a). LPA also produced a time-dependent increase in DDR2 expression in ES2 cells, while the maximum expression of DDR2 in A2780 cells was observed at 12 h with LPA treatment (Figure 1b). Likewise, immunofluorescence analysis showed the increased expression of DDR2 by LPA (Figure 1c). Importantly, the DDR2 siRNA significantly attenuated LPA-induced ovarian cancer cell 2D invasion (Figure 1d), as well as migration (Figure 1e and Figure S1a). In addition, the silencing of DDR2 expression markedly decreased LPA-induced cancer cell invasive foci formation and growth on 3D Matrigel (Figure 1f and Figure S1b) and 3D Matrigel-coated transwell chambers (Figure 1g). Together, these data indicate that DDR2 expression is important in LPA-induced ovarian cancer cell invasion.

### 2.2. ATX Induces DDR2 Expression

**Figure 2:**
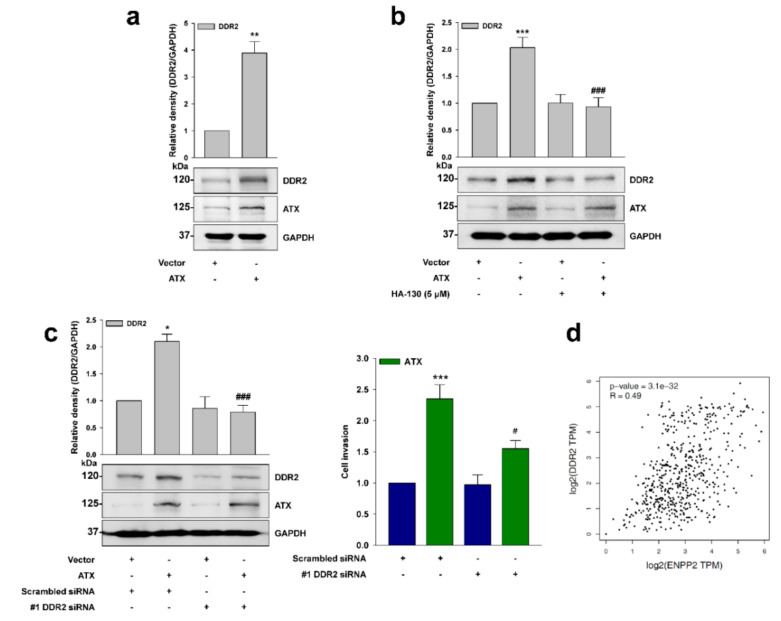
ATX induces DDR2 expression. (**a**) ES2 cells were transfected with the indicated vectors and then the resulting cell lysates were subjected to immunoblot analysis (mean ± s.d. ** *p* < 0.01 versus control). (**b**) ES2 cells were transfected with the indicated vectors and then treated with HA-130 (5 μM) for 24 h before being subjected to immunoblot analysis (mean ± s.d. *** *p* < 0.001 versus control and ### *p* < 0.001 versus ATX overexpression). (**c**) ES2 cells were co-transfected with the indicated vectors and siRNAs prior to invasion assay (mean ± s.d. * *p* < 0.05 and *** *p* < 0.001 versus control, # *p* < 0.05 and ### *p* < 0.001 versus ATX overexpression). (**d**) Correlation between *DDR2* and *ENPP2* gene expression in ovarian cancer. Empty vectors and scrambled siRNA were used as controls. Representative results were presented from at least three independent experiments with similar results.

Since ATX is an ectoenzyme producing LPA extracellularly [29], we next determined the effect of ATX on DDR2 expression. Ectopic expression of ATX upregulated DDR2 expression (Figure 2a). Conversely, treatment of the cells with a selective pharmacological inhibitor of ATX, HA-130, markedly inhibited ATX-induced DDR2 expression (Figure 2b). In addition, the silencing of DDR2 expression abolished ATX-induced ovarian cancer cell invasion (Figure 2c). Moreover, the ovarian cancer patient profiles of the GEPIA dataset showed a significant positive correlation of ATX (*ENPP2*) expression with DDR2 (*DDR2*) expression (*r* = 0.49, *p* = 3.1 × 10^−32^) (Figure 2d). Therefore, these data imply that DDR2 is important for ATX- and, thereby, LPA-induced ovarian cancer cell invasion.

### 2.3. LPA Upregulates Twist1 Expression

**Figure 3:**
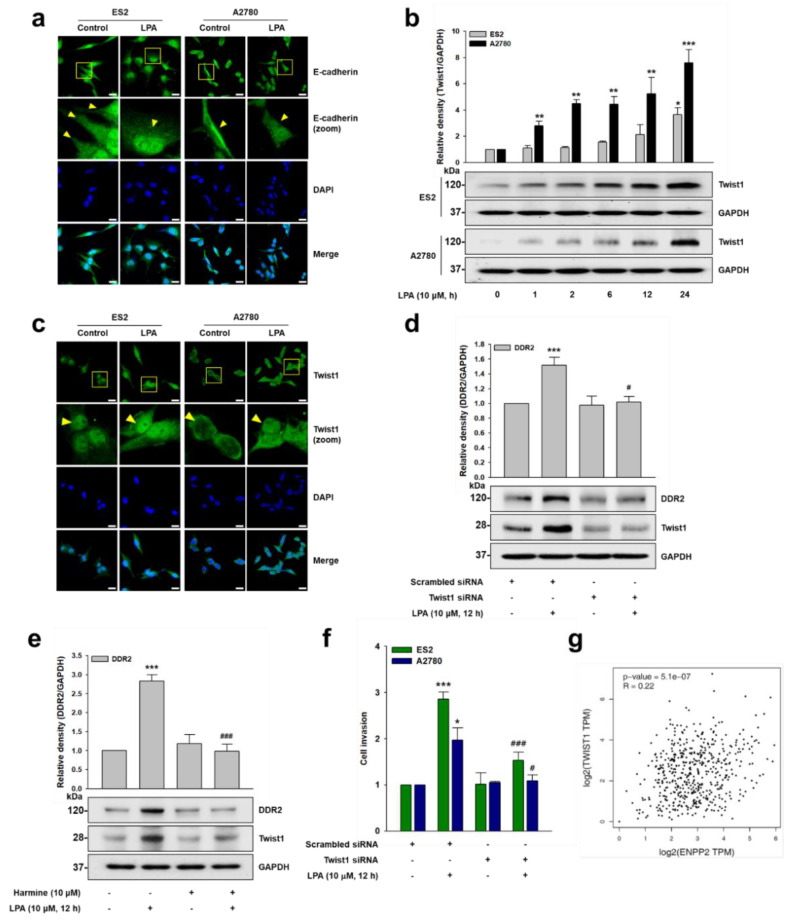
LPA upregulates Twist1 expression. (**a**,**c**) The serum-starved cells were stimulated with LPA (10 μM) for 12 h, and then the expression of (**a**) E-cadherin and (**c**) Twist1 was visualized via immunofluorescence (green: E-cadherin or Twist1, and blue: Nucleus). Original magnification: ×400; scale bar: 20 μm. (**b**) The serum-starved cells were stimulated with LPA (10 μM) for the indicated times, before immunoblotting (mean ± s.d. * *p* < 0.05, ** *p* < 0.01 and *** *p* < 0.001 versus control). (**d**) ES2 cells were transfected with the indicated siRNAs and then stimulated with LPA (10 μM) for 12 h, before immunoblotting (mean ± s.d. *** *p* < 0.001 versus control and # *p* < 0.05 versus LPA treatment only). (**e**) The serum-starved ES2 cells were pretreated with harmine (10 μM) for 1 h and then stimulated with LPA (10 μM) for 12 h, before immunoblotting (mean ± s.d. *** *p* < 0.001 versus control and ### *p* < 0.001 versus LPA treatment only). (**f**) The cells were transfected with the indicated siRNAs, followed by stimulation with LPA (10 μM), and then subjected to invasion assay (mean ± s.d. * *p* < 0.05 and *** *p* < 0.001 versus control, # *p* < 0.05 and ### *p* < 0.001 versus LPA treatment only). (**g**) Correlation between *TWIST1* and *ENPP2* gene expression in ovarian cancer. Scrambled siRNAs were used as controls. Representative results were presented from at least three independent experiments with similar results.

Previously, LPA was reported to induce EMT in ovarian cancer [30,31]. In addition, Twist1 was shown to induce DDR2 expression in ovarian cancer [21]. In order to identify the role of Twist1 in LPA-induced DDR2 expression, we first determined whether LPA induces EMT in our system. Stimulation of the cells with LPA markedly reduced E-cadherin expression (Figure 3a). We next determined whether LPA induces Twist1 and, thereby, DDR2 expression. Immunoblotting (Figure 3b) and immunofluorescence (Figure 3c) data clearly showed that LPA induces Twist1 expression. However, the silencing of Twist1 expression (Figure 3d), or treatment of the cells with a selective pharmacological inhibitor of Twist1, harmine (Figure 3e), dramatically downregulated LPA-induced DDR2 expression. Furthermore, the silencing of Twist1 expression significantly inhibited LPA-induced ovarian cancer cell invasion (Figure 3f). Interestingly, the ovarian cancer patient profiles appeared to positively correlate with ATX (*ENPP2*) and Twist1 (*TWIST1*) expression in the GEPIA dataset (*r* = 0.22, *p* = 5.1 × 10^−7^) (Figure 3g). Therefore, these data indicate that LPA induces ovarian cancer cell EMT, and that an EMT-TF Twist1 mediates LPA-induced DDR2 expression, as well as ovarian cancer cell invasion.

### 2.4. HIF-1α Is Critical for Twist1 Expression

**Figure 4:**
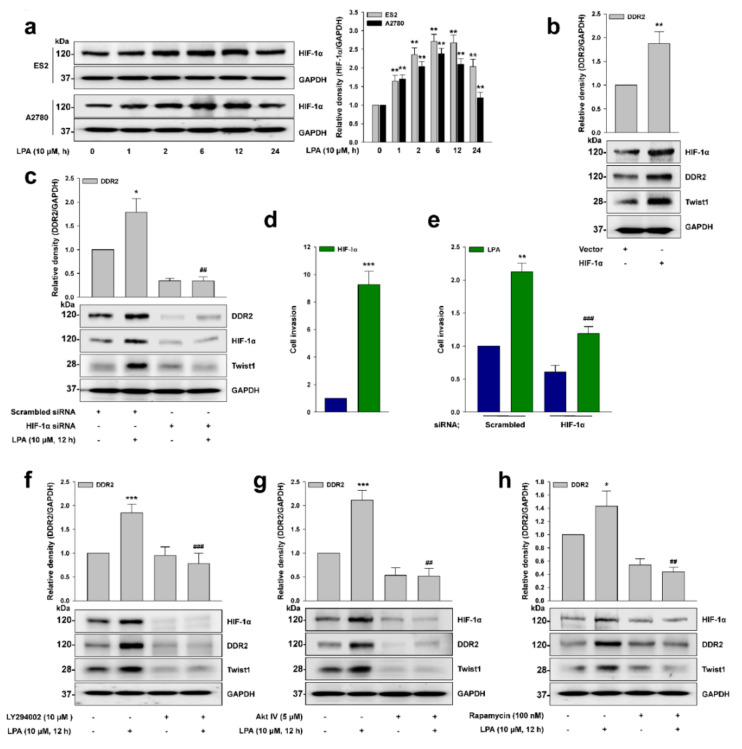
HIF-1α is critical for Twist1 expression. (**a**) The serum-starved cells were stimulated with LPA (10 μM) for the indicated times, before immunoblotting (mean ± s.d. ** *p* < 0.01 versus control). (**b**) ES2 cells were transfected with the indicated vectors, and the lysates were subjected to immunoblot analysis (mean ± s.d. ** *p* < 0.01 versus control). (**c**) ES2 cells were transfected with the indicated siRNAs and then stimulated with LPA (10 μM) for 12 h, before immunoblotting (mean ± s.d. * *p* < 0.05 versus control and ## *p* < 0.01 versus LPA treatment only). (**d**) ES2 cells were transfected with the indicated vectors for invasion assay (mean ± s.d. *** *p* < 0.001 versus control). (**e**) ES2 cells were transfected with the indicated siRNAs, followed by stimulation with LPA (10 μM) for invasion assay (mean ± s.d. ** *p* < 0.01 versus control and ### *p* < 0.001 versus LPA treatment only). (**f**–**h**) The serum-starved ES2 cells were pretreated with the indicated inhibitors for 1 h, followed by stimulation with LPA (10 μM) for 12 h, before immunoblotting (mean ± s.d. * *p* < 0.05 and *** *p* < 0.001 versus control, ## *p* < 0.01 and ### *p* < 0.001 versus LPA treatment only). Empty vectors and scrambled siRNAs were used as controls. Representative results were presented from at least three independent experiments with similar results.

Given that LPA induces HIF-1α expression [12], and that DDR2 expression is correlated with HIF-1α expression [25], we next explored whether HIF-1α is important for LPA-induced DDR2 expression. LPA upregulated HIF-1α expression in the tested cancer cells (Figure 4a). We also observed the increased expression of DDR2 and Twist1 by HIF-1α (Figure 4b), while the silencing of HIF-1α expression dramatically inhibited LPA-induced Twist1 and DDR2 expression (Figure 4c). Consistently, we noted that HIF-1α induced cancer cell invasion (Figure 4d), and that the silencing of HIF-1α expression significantly inhibited LPA-induced cancer cell invasion (Figure 4e). In addition, pretreatment of the cells with selective pharmacological inhibitors of phosphoinositide 3-kinase (PI3K, LY294002, Figure 4f), Akt (Akt IV, Figure 4g), and mTOR (Rapamycin, Figure 4h) drastically inhibited LPA-induced HIF-1α and DDR2 expression. Collectively, these results indicate that LPA induces HIF-1α expression through the PI3K/Akt/mTOR signaling pathways, which promotes Twist1 and, thereby, DDR2 expression.

### 2.5. DDR2 Mediates LPA-Induced MT1-MMP Expression

**Figure 5:**
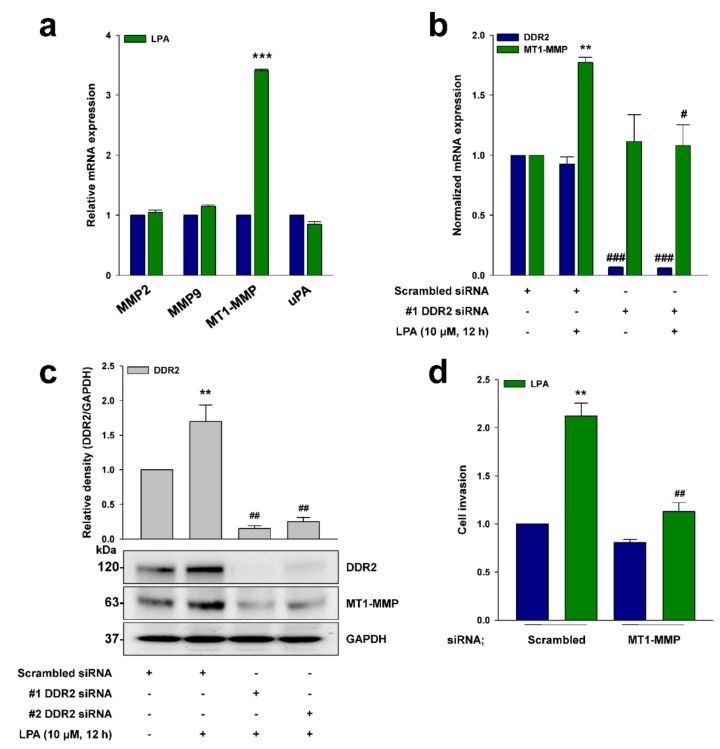
DDR2 mediates LPA-induced MT1-MMP expression. (**a**) The serum-starved ES2 cells were stimulated with LPA (10 μM) for 12 h, before qRT-PCR analysis (mean ± s.d. *** *p* < 0.001 versus control). (**b**) ES2 cells were transfected with the indicated siRNAs and then stimulated with LPA (10 μM) for 12 h, before qRT-PCR analysis (mean ± s.d. ** *p* < 0.01 versus control, # *p* < 0.05 and ### *p* < 0.001 versus LPA treatment only). (**c**) ES2 cells were transfected with the indicated siRNAs and then stimulated with LPA (10 μM) for 12 h, before immunoblotting (mean ± s.d. ** *p* < 0.01 versus control and ## *p* < 0.01 versus LPA treatment only). (**d**) ES2 cells were transfected with the indicated siRNAs and then stimulated with LPA (10 μM) for invasion assay (mean ± s.d. ** *p* < 0.01 versus control and ## *p* < 0.01 versus LPA treatment only). Scrambled siRNAs were used as controls. Representative results were presented from at least three independent experiments with similar results.

Previously, DDR2 was reported to induce ovarian cancer cell invasion through the expression of various proteolytic enzymes [21]. In order to identify which proteolytic enzymes are responsible for LPA-induced ovarian cancer cell invasion, we treated the cells with LPA, and observed a dramatic increase of MT1-MMP mRNA expression (Figure 5a). In addition, the silencing of DDR2 expression completely abolished the LPA-induced MT1-MMP transcription (Figure 5b) and protein expression (Figure 5c). Finally, the silencing of MT1-MMP expression abolished the LPA-induced ovarian cancer cell invasion (Figure 5d). Therefore, these data strongly suggest that MT1-MMP induced via DDR2 is important for LPA-induced ovarian cancer cell invasion.

## 3. Discussion

Produced extracellularly by the ectoenzyme ATX, LPA has been implicated in a plethora of pathophysiological events, including cancer invasion and metastasis. Given that elevated levels of LPA were observed in the serum and ascites of ovarian cancer patients [10], the production of LPA and its downstream signaling have been recognized as being among the major driving factors for ovarian cancer. Although LPA interacts with its already-known GPCRs, intracellular signaling of GPCRs cooperates with other types of receptor. Indeed, LPA-activated GPCRs have been shown to transactivate with EGFR to increase ovarian cancer cell invasiveness [26]. In the present study, we identify the underlying mechanism by which LPA induces the expression of another receptor tyrosine kinase, DDR2, which is closely associated with ovarian cancer progression.

ATX was initially identified as an autocrine motility factor with phosphodiesterase activity [32,33]. However, two independent groups discovered that ATX has lysophosphodiesterase D activity, allowing it to produce LPA extracellularly [29,34]. We in the present study provide evidence that ATX, and thereby LPA, aggravates ovarian cancer cell invasion through DDR2 expression. First, both ATX and LPA upregulated DDR2 expression. In addition, pretreatment of the cells with a selective pharmacological inhibitor of ATX ablated ATX-induced DDR2 expression and ovarian cancer invasion. Second, the expression of ATX is closely correlated with that of DDR2 in ovarian cancer patients. Lastly, LPA produced MT1-MMP, which has been shown to be a downstream proteolytic factor of DDR2 for ovarian cancer cell invasion.

DDRs are distinct types of RTK activated by collagens instead of growth factors, associated with cell growth and metastasis in various cancers [35]. In addition, a recent study emphasizes the clinical significance of DDR2 as a potential marker for cancers, showing that targeting DDR2 potentiates anti-PD-1 clinical response [36]. Mechanistically, DDR2 is closely associated with EMT. DDR2 stabilizes Snail and subsequent proteolytic enzyme production [19]. Recently, Grither et al. demonstrated that Twist1 is implicated in DDR2 upregulation and ovarian cancer cell metastasis [21]. In the present study, we demonstrate that LPA upregulates Twist1 expression through a PI3K/Akt/mTOR and HIF-1α signaling cascade. Although LPA has been known to induce EMT through EZH2 [37], Slug [31], and β-catenin [30], the present study is the first to demonstrate LPA-induced Twist1 expression. It is noteworthy that LPA induces DDR2 protein expression but not mRNA expression. In view of a previous report claiming that heat shock protein 47 (HSP47) regulates DDR2 protein stability [38], the effects of LPA or Twist1 on HSP47 and, thereby, DDR2 protein stability are under current investigation.

**Figure 6:**
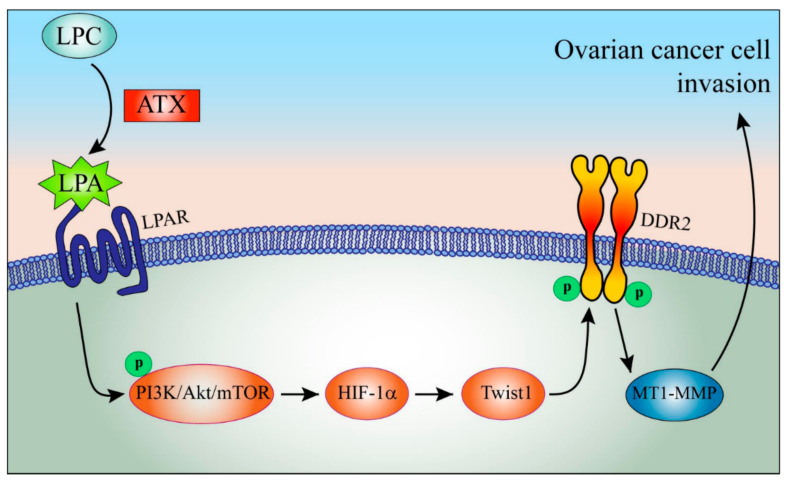
The schematic working model of LPA-induced DDR2 expression and ovarian cancer cell invasion. LPA induces DDR2 expression through activation of PI3K/Akt/mTOR/HIF-1α/Twist1 signaling, aggravating ovarian cancer cell invasion.

Accumulating evidence shows the association of LPA with HIF-1α. LPA was shown to induce HIF-1α in several cancers, including ovarian cancer [12]. In addition, hypoxic conditions enhance the responsiveness of LPA in ovarian cancer [39,40,41]. Moreover, the essential role of HIF-1α was proposed in LPA-induced ovarian cancer EMT through Gi and Src [31]. Our present data show the critical role of PI3K/Akt/mTOR signaling in LPA-induced HIF-1α expression, which is in line with previous reports showing the LPA-induced stabilization of HIF-1α through PI3K [12,42]. The schematic signaling pathway of LPA-induced DDR2 expression depicts the upregulation of DDR2 by LPA through coordinate activation of the PI3K/Akt/mTOR/HIF-1α signaling pathways and Twist1 expression to augment ovarian cancer cell invasion (Figure 6).

Collectively, our results show for the first time that LPA induces DDR2 expression and consequent ovarian cancer cell invasion through the HIF-1α and Twist1 signaling axes, reinforcing the significance of LPA and the transactivation between GPCR and RTK in ovarian cancer progression. Therefore, PI3K, Akt, HIF-1α, Twist1, and DDR2 may serve as effective targets to attenuate the invasion and metastasis of ovarian cancer cells.

## 4. Material and Methods

### 4.1. Reagents

Harmine was acquired from Sigma-Aldrich (St. Louis, MO, USA). LPA was acquired from Avanti Polar Lipids (Alabaster, AL, USA). All used reagents were of the purest grade available. LY294002, AKT IV, and Rapamycin were obtained from Calbiochem (San Diego, CA, USA).

### 4.2. Cell Culture

All ovarian cancer cell lines were purchased from the American Type Culture Collection (Manassas, VA, USA). The ES2 and A2780 cells were maintained in RPMI 1640 supplemented with 10% fetal bovine serum (FBS) and 1% penicillin/streptomycin. All cells were incubated at 37 °C under 5% CO_2_ in a humidified incubator.

### 4.3. Small Interfering RNA (siRNA) and Plasmid Transfection

The cells were transfected using Lipofectamine3000 or RNAiMAX (Invitrogen, Carlsbad, CA, USA) as manual description. The siRNAs of Twist1, HIF-1α, and #1 and #2 DDR2 were obtained from Sigma-Aldrich. LPAR1 siRNA was obtained from Dharmacon (Lafayette, CO, USA). Scrambled siRNAs were obtained from Invitrogen and used as controls. cDNAs for pcDNA4-ATX and pcDNA3-HIF-1α were purchased from Invitrogen and Addgene (Boston, MA, USA), respectively.

### 4.4. Immunoblotting

The cell lysates were prepared as described previously [37]. Antibodies for DDR2, Twist1, HIF-1α, and MT1-MMP were obtained from Cell Signaling Technology (Danvers, MA, USA). An ATX antibody was purchased from Abcam (Cambridge, MA, USA). Antibodies for E-cadherin and glyceraldehyde 3-phosphate dehydrogenase (GAPDH) were purchased from Santa Cruz Biotechnology Inc. (Santa Cruz, CA, USA). Anti-rabbit and anti-mouse secondary antibodies were obtained from Thermo Fisher Scientific Inc. (Rockford, IL, USA). The immunoblotting bands were visualized by ECL (Thermo Fisher Scientific Inc., Rockford, IL, USA) using Image Quant 400 (GE Healthcare, Buckinghamshire, UK).

### 4.5. Quantitative Real-Time PCR (qRT-PCR)

Total RNA was reverse transcribed into cDNA using a Moloney murine leukemia virus (M-MLV, Promega, Madison, WI, USA), as previously described [37]. The cDNA was then subjected to PCR amplification with primer sets for DDR2, MMP2, MMP9, MT1-MMP, uPA, and GAPDH: DDR2 forward (5′-CTC CCA GAA TTT GCT CCA G-3′); DDR2 reverse (5′-GCC ACA TCT TTT CCT GAG A-3′); MMP2 forward (5′-ATG ACA GCT GCA CCA CTG AG-3′); MMP2 reverse (5′-AGT TCC CAC CAA CAG TGG AC-3′); MMP9 forward (5′- GTG CCA TGT AAA TCC CCA CT-3′); MMP9 reverse (5′-CTC CAC TCC TCC CTT TCC TC-3′); MT1-MMP forward (5′-TTG GAC TGT CAG GAA TGA GG-3′); MT1-MMP reverse (5′-GCA GCA CAA AAT TCT CCG TG-3′); uPA forward (5′-GTG GCC AAA AGA CTC TGA GG-3′); uPA reverse (5′-GCC GTA CAT GAA GCA GTG TG-3′); GAPDH forward (5′-ACA GTC AGC CGC ATC TTC TT-3′); and GAPDH reverse (5′-ACG ACC AAA TCC GTT GAC TC-3′). The GAPDH gene was utilized as a control for calculating dCt value. The qRT-PCR results were analyzed using the 2-(ddCt) method.

### 4.6. In Vitro Invasion Assay

In vitro cancer cell invasion assays were performed with an invasion assay kit with Matrigel-coated membranes (BD Biosciences, San Jose, CA, USA), as described previously [43]. Volumes of 5 × 10^5^ cells per well were injected into the upper chamber. To the lower chamber, we added the serum-free conditioned medium, with or without reagent. After incubation for 16–18 h at 37 °C, the membranes were fixed and stained using a Diff-Quik kit (Dade Behring, Inc., Newark, DE, USA). The average numbers of four random microscopic fields (×200) were recorded in each experiment.

### 4.7. Immunofluorescence Assay

Immunofluorescence assays were performed as described previously [44]. Antibodies for E-cadherin and Twist1 were obtained from Santa Cruz Biotechnology Inc. A DDR2 antibody was obtained from Abcam. The cells were incubated with Cy3-conjugated anti-rabbit IgG and Cy3-conjugated anti-mouse IgG antibodies (Jackson Immunoresearch, West Grove, PA, USA). The nuclei of the cells were stained with 4′,6-Diamidino-2-Phenylindole (DAPI, Invitrogen). The samples were analyzed via confocal microscopy (LSM710, Carl Zeiss, Jena, Germany).

### 4.8. Wound Healing Migration Assay

Wound healing migration assays were performed as described previously [45]. Photographic images were snapped immediately after scraping and after 12 h in the same locations. Wound closure rate was determined using image J (Bethesda, MD, USA). Cells were examined using a light microscope.

### 4.9. Three-Dimensional (3D) Matrigel Invasion Assay

3D Matrigel invasion assays were performed and analyzed as described previously [46,47,48,49], with some modifications. After labeling with DiI (Thermo Fisher Scientific, Waltham, MA, USA), ES2 cells (3 × 10^4^ cells/mL) were mixed in 200 μL of medium supplemented with 0.2% FBS and plated on gels. The low 24-well plates were filled with 10% FBS supplemented medium. After 5 days, the embedded gel was sectioned, and the cells were analyzed via fluorescence confocal microscopy. The distance between the invaded cells was measured from eight different positions and calculated using the ZEN blue edition program of Carl Zeiss Microscopy GmbH. The distance in μm was calculated as described previously [50].

### 4.10. 3D Matrigel Morphogenesis Assay

3D Matrigel morphogenesis assays were executed as described previously [51]. The 3 × 10^4^ cells/mL cells were resuspended in 400 μL medium and supplemented with 2% Matrigel. Cells were grown for 7 days, and the media were changed every 2 days. Cells were monitored every day and examined using a light microscope.

### 4.11. GEPIA Database Analysis

The Gene Expression Profiling Interactive Analysis (GEPIA; http://gepia.cancer-pku.cn/index.html, accessed 13 August 2019) is a web server designed to perform customizable analyses of the RNA sequencing expression data of 426 ovarian cancer and 88 normal samples from the Cancer Genome Atlas (TCGA) and the Genotype-Tissue Expression Project (GTEx). We analyzed the correlations between expression of *ENPP2* and *DDR2* or *TWIST1* in our study through related modules in GEPIA. Spearman’s rank correlation analysis was used to determine the correlation coefficient.

### 4.12. Statistical Analyses

Data are presented as the mean ± standard deviation (s.d.). Statistical analysis was assessed with SigmaPlot software (SYSTAT Software, San Jose, CA, USA) using Student’s *t*-test. Differences between three or more groups were estimated by analysis of variance, followed by Bonferroni multiple comparison tests.

## Supplementary Materials

Supplementary materials can be found at https://www.mdpi.com/article/10.3390/ijms22105374/s1, Figure S1: The silencing of DDR2 expression inhibits LPA-induced ovarian cancer cell aggressiveness.
