# Supplementary material for: Discoidin Domain Receptor 2 Mediates Lysophosphatidic Acid-Induced Ovarian Cancer Aggressiveness
Source: Int J Mol Sci. 2021 May 20;22(10):5374. doi: 10.3390/ijms22105374 (PMC8160857; doi:10.3390/ijms22105374)
Supplement: Supplementary file 1 [file ijms-22-05374-s001.zip › ijms-1218776-supplementary.pdf]

Supplementary Figure S1

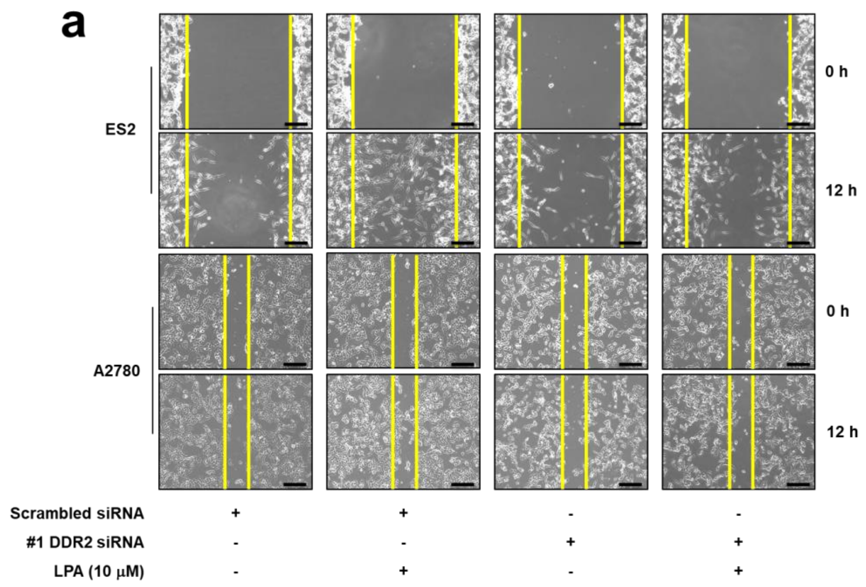

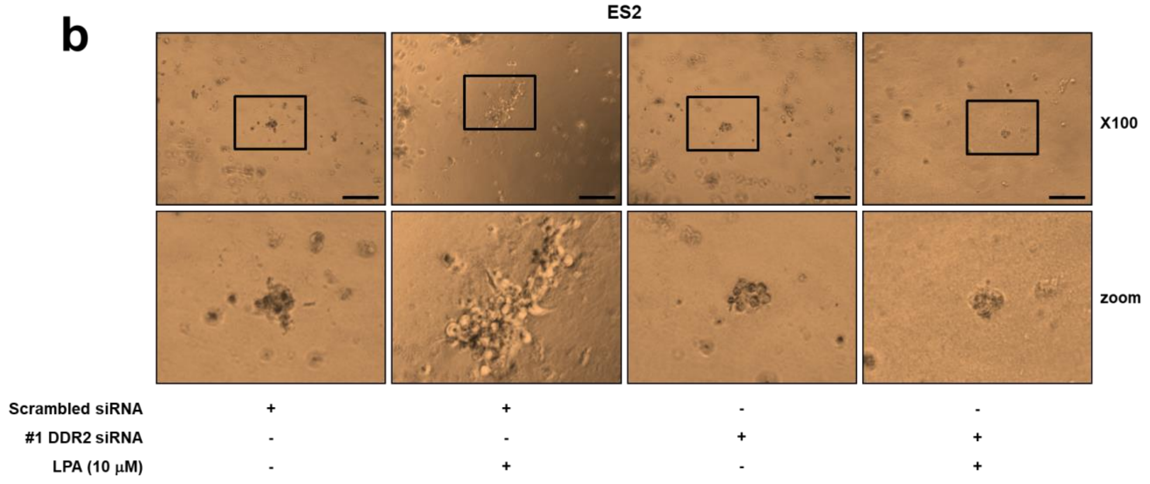

Figure S1. The silencing of DDR2 expression inhibits LPA-induced ovarian cancer cell aggressiveness. (**a**) The cells were transfected with indicated siRNAs and then stimulated with LPA (10 μM) for wound healing migration assay. Original magnification, ×100; scale bar, 100 μm. (**b**) ES2 cells were cultured on 3D Matrigel for 7 days followed by counting branches. Original magnification, ×100; scale bar, 100 μm. Results were triplicates from three independent experiments with similar results.
